# Synthesis and Crystal Structure of the Zintl Phases NaSrSb, NaBaSb and NaEuSb

**DOI:** 10.3390/ma16041428

**Published:** 2023-02-08

**Authors:** Yi Wang, Svilen Bobev

**Affiliations:** Department of Chemistry and Biochemistry, University of Delaware, Newark, DE 19716, USA

**Keywords:** antimonides, crystal structure, thermoelectrics, topological insulator, Zintl phases

## Abstract

This work details the synthesis and the crystal structures of the ternary compounds NaSrSb, NaBaSb and NaEuSb. They are isostructural and adopt the hexagonal ZrNiAl-type structure (space group *P*$\bar{6}$2*m*; Pearson code *hP*9). The structure determination in all three cases was performed using single-crystal X-ray diffraction methods. The structure features isolated Sb^3–^ anions arranged in layers stacked along the crystallographic *c*-axis. In the interstices, alkali and alkaline-earth metal cations are found in tetrahedral and square pyramidal coordination environments, respectively. The formal partitioning of the valence electrons adheres to the valence rules, i.e., Na^+^Sr^2+^Sb^3–^, Na^+^Ba^2+^Sb^3–^ and Na^+^Eu^2+^Sb^3–^ can be considered as Zintl phases with intrinsic semiconductor behavior. Electronic band structure calculations conducted for NaBaSb are consistent with this notion and show a direct gap of approx. 0.9 eV. Additionally, the calculations hint at possible inverted Dirac cones, a feature that is reminiscent of topological quantum materials.

## 1. Introduction

Compounds formed between the alkali metals (*A*), the alkaline earth metals (*AE*), and the group 15 elements (P, As, Sb, Bi, referred to as pnictogens, hereafter denoted as *Pn*) have been studied for over 50 years. A variety of compositions are known to date, with the most recurring being the equiatomic *A*–*AE*–*Pn* structures [1]. Among them, several different structure types are reported, in which a relatively straightforward dependence on the atomic size values could be observed. For example, with smaller *A*- and *AE*-atoms, the cubic half-Heusler structure (viz. LiMgP) [2] and the hexagonal LiGaGe structure (viz. LiBeSb) [3] are adopted. With intermediate size *A*- and *AE*-atoms, the tetragonal PbClF or Cu_2_Sb structure (viz. NaMgAs and KCaBi) [4,5], and hexagonal ZrBeSi or KZnP structure (viz. NaBeSb and BaLiP) [6,7] are adopted. Compounds containing Li in addition to the aforementioned structures appear to most strongly favor the orthorhombic TiNiSi structure (viz. CaLiSb and SrLiAs) [8,9] as well as the hexagonal BaLiSi structure (viz. BaLiAs) [10]. As the average size of the *A*- and *AE*-atoms becomes larger, the hexagonal ZrNiAl or Fe_2_P structure becomes more common, as exemplified by NaSrP [7], NaSrAs [11], NaBaP [12] and NaBaBi [13]. The latter four are the only known pnictides with *A*- and *AE*-cations, so far, that crystallize isostructurally with the ZrNiAl type.

Our group has had a long-standing interest in the solid-state chemistry of pnictides [14,15]. Over the years, we have conducted studies on related ternary *A*–*AE*–*Pn* compounds too. The focus of our attention was on the systems Li–*AE*–*Pn*, where *AE* not only represents the alkaline earth metals Ca, Sr and Ba, but also the nominally divalent rare earth metals Eu and Yb [16]. As a result, several new compounds were synthesized and structurally characterized [16]. Later, the ongoing studies were expanded towards Na–*AE*–*Pn* and the more complex systems Li–*AE*–*E*–*Pn*, Li–*AE*–*Tr*–*Pn*, Na–*AE*–*E*–*Pn* and Na–*AE*–*Tr*–*Pn* (*E* = group 12, *Tr* = group 13) [17,18,19,20,21].

In this paper, we report the synthesis and the structural analysis of three ternary antimonides that were encountered along the way, namely NaSrSb, NaBaSb and NaEuSb. The crystal structures of all of these new compounds were established from single-crystal X-ray diffraction methods. The structures are new additions to the large ZrNiAl (or Fe_2_P) structural family. There is a full cation ordering in each case, indicating that the electron count is consistent with the valence rules, i.e., Na^+^Sr^2+^Sb^3–^, Na^+^Ba^2+^Sb^3–^ and Na^+^Eu^2+^Sb^3–^ are Zintl phases [22]. The electronic structure of NaBaSb, chosen as a representative, is also presented and briefly discussed.

## 2. Results and Discussion

Details of the data collection and selected crystallographic parameters are summarized in Table 1.

As mentioned previously, many intermetallic compounds with equiatomic compositions are known and crystallize with over 25 structure types. NaSrSb, NaBaSb and NaEuSb are isotypic and crystallize with the structure type that is known as either ZrNiAl or Fe_2_P (Pearson symbol *hP*9) [1]. The only other known pnictides with *A*- and *AE*-cations and a simple 1:1:1 ratio that form with the same structure are NaSrP [7], NaSrAs [11], NaBaP [12] and NaBaBi [13].

**Figure 1:**
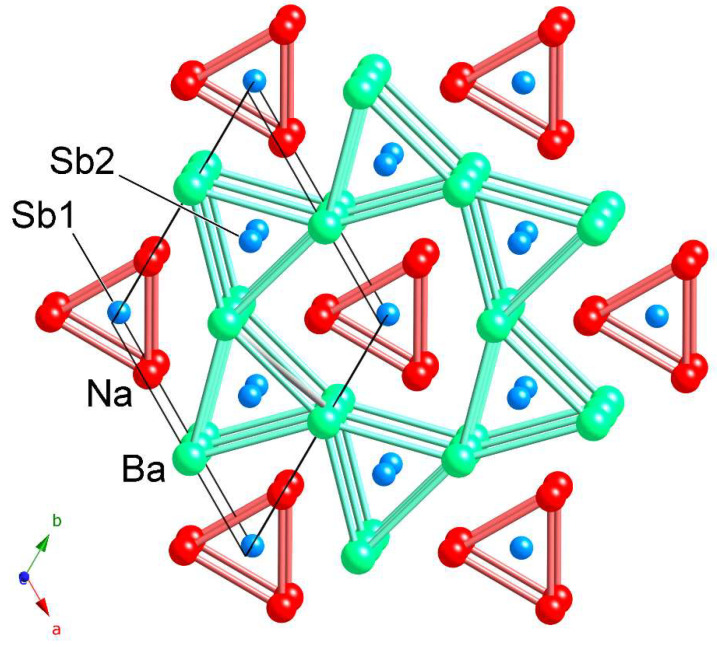
The hexagonal crystal structure of NaBaSb projected down the *c*-axis. The representation emphasizes the arrangement of trigonal prisms made by Na atoms (red) and Ba atoms (light green), which host the Sb atoms (light blue). The unit cell is outlined and the symmetry-unique atoms are labeled.

The structure is hexagonal and has the non-centrosymmetric space group *P*$\bar{6}$2*m* (no. 189). The prototype structure has been discussed at length in many other publications, including review articles [23,24]; therefore, we will cover it only briefly. For the sake of simplicity, NaBaSb was chosen as a representative structure for the following presentation. The structure is drawn schematically in Figure 1.

There are four independent crystallographic positions in the asymmetric unit (Table 2), including one sodium, one barium (or strontium or europium), and two antimony sites, all in special positions. The structure can be described in multiple ways, and can be related to other simpler structures—for example, the similarities between the AlB_2_ and Fe_2_P (ZrNiAl) structures are shown schematically in the Supplementary Materials sectioin, and a full crystallographic treatment of the structural relationship can be found in [23].

The most common way of visualization of the crystal structure is as an array of trigonal prisms made by Na and Ba atoms, organized as shown in Figure 1. The prisms are stacked along the *c*-direction, with the Ba_6_ prisms sharing common edges to form six-membered “channels” that host the Na_6_ prisms. This description is an over-simplification since the three open rectangular faces of the prisms are capped, which allows them to be intergrown, rather than isolated as in the schematic. The larger barium atoms (relative to Sr and Eu) expand the triangular arrangement in the *ab*-plane, as evident from the distances tabulated in Table 3. The two crystallographically independent antimony atoms fill the Na_6_ and Ba_6_ trigonal prisms, respectively. The Sb atoms are located at the centers of each prism, which positions them very much apart; there are no direct Sb–Sb interactions. Thus, NaBaSb can be treated as a salt-like Na^+^Ba^2+^Sb^3–^ compound, i.e., a Zintl phase [22].

**Figure 2:**
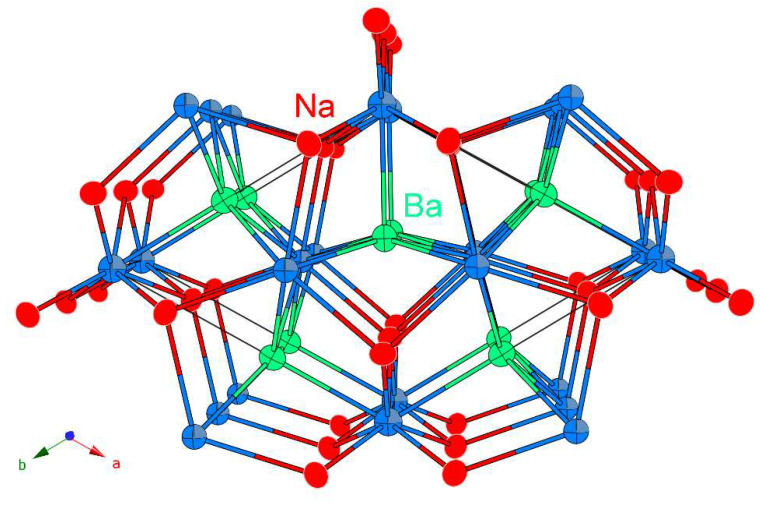
The crystal structure of NaBaSb shown with the refined anisotropic displacement parameters for all atoms (95% probability level). The Ba atoms (light green) are coordinated to five Sb atoms (light blue) in a square-pyramidal fashion. The Na atoms (red) are tetrahedrally coordinated to four Sb atoms.

As a complementary description, one can visualize the structure as hexagonal sheets of Sb1 at *z* = 0 and Sb2 at *z* = ½, with the Na and Ba occupying the interstitials. This structural view is emphasized in Figure 2. In this viewpoint, which is akin to the description of many ionic salts, the Na^+^ cations can be seen as located in tetrahedral coordination by the Sb^3–^ anions, filling half of the available tetrahedral holes (CN 4). The Ba^2+^ cations have different size and charge; therefore, one should expect a different type of coordination environment. Indeed, Ba^2+^ cations are coordinated square-pyramidally by the Sb^3–^ anions, filling half of the available pyramidal holes (CN 5). Tetrahedra and square pyramids share common vertices and edges.

Figure 2 also shows all atoms with their refined anisotropic displacement parameters. The ellipsoids have indeed very similar principal dimensions and are rather spherical; no enlarged atomic displacements are exhibited, which is a testament to a highly ordered crystallographic structure.

Another noteworthy crystallographic feature is the slightly smaller (ca. 2%) unit cell volume of NaEuSb compared to that of NaSrSb (Table 1). This is indirect evidence for the divalent state of Eu in this material, since Eu^2+^ and Sr^2+^ have very similar ionic radii [25]. On the other hand, Eu^3+^ is much smaller than the alkaline earth metal cation, which should be manifested in a much larger reduction in the unit cell volume compared to what is currently observed. To date, phase-pure NaEuSb samples are not available, and the above supposition could not be experimentally validated via magnetic susceptibility measurements.

**Figure 3:**
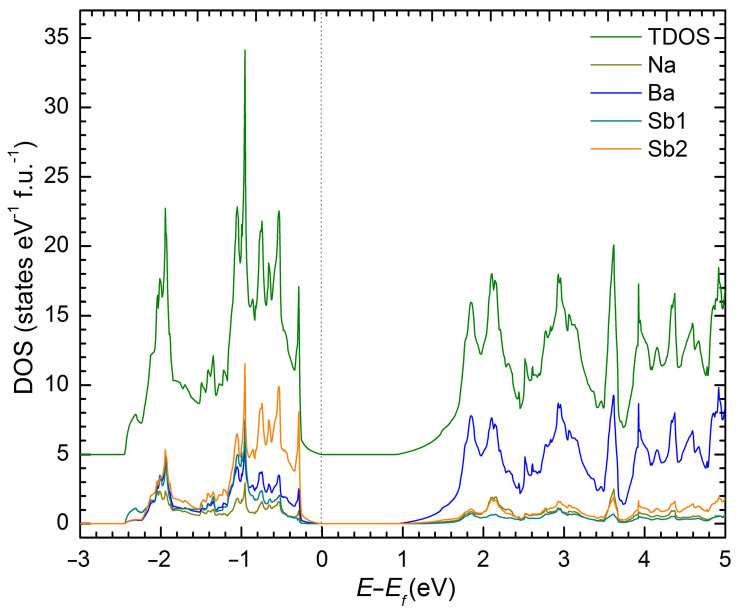
The stacked atom-projected electronic density of states (DOS) for NaBaSb.

Since NaBaSb is a compound that can be conceptually derived from the binary Na_3_Sb Zintl phase ([Na^+^]_3_Sb^3–^) by replacing two Na^+^ cations with one Ba^2+^ cation, and since Na_3_Sb and Na_3_Bi have been extensively studied for their topological properties [26,27,28], it was deemed important to gain some insights into the electronic band structure of this new material. Therefore, we performed a computational analysis using density functional theory, as implemented in the LMTO method [29,30]. The results are graphically summarized in Figure 3 and show the emergence of a bandgap of approx. 0.9 eV. The majority of the states near the bottom of the conduction band are contributed by Ba orbitals. The states close to the valence band maximum are contributed mainly by Sb orbitals, with minor contributions from Ba and Na. The minor overlap of Ba and Sb evidenced in the valence band (states between –1.5 eV and 0 eV; recall that we plot *E*–*E*_F_ and zero is the Fermi level) suggests that there are some covalent features of the interactions between Ba and Sb.

**Figure 4:**
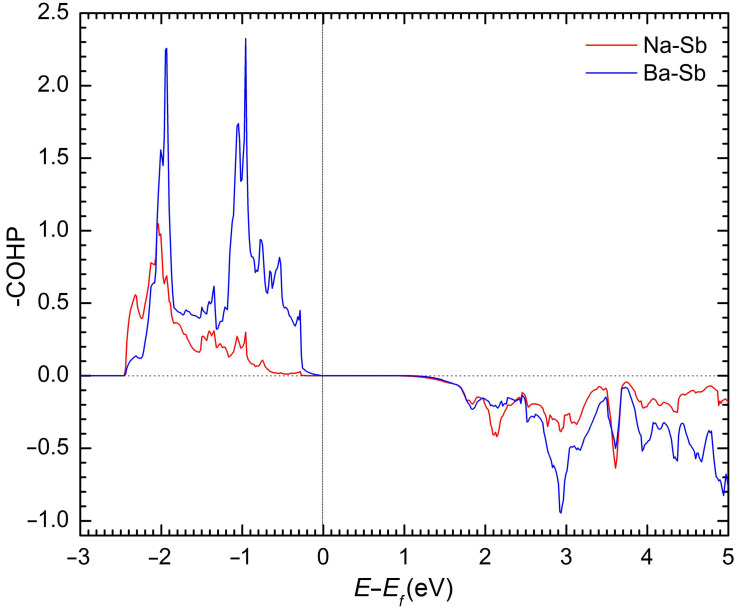
Crystal orbital Hamilton population curves for Na–Sb and Ba–Sb in NaBaSb.

To further interrogate the subtle covalent features of Ba–Sb and Na–Sb bonding, we next consider their crystal orbital Hamilton population curves (COHP) [31], plotted in Figure 4. Based on them, one can conclude that the atomic interactions are optimized at the Fermi level. No antibonding states for both Na–Sb and Ba–Sb interactions can be observed below the Fermi level. The observations above suggest that although NaBaSb fits nicely within the classical description of a Zintl phase [22], some degree of covalency of the interactions for the presumed “cations” should also be expected.

It should also be noted that the estimated bandgap of 0.9 eV in NaBaSb is much larger than the reported 0.23 eV in NaBaBi [32]. Such an observation may likely be due to the overestimation of the bandgap by the LMTO-ASA code. In any case, it is reasonable to expect that the real bandgap will not vary much from that of the counterpart bismuthide.

In the context of the presented DOS and COHP results, it is instructive to draw attention to the fact that NaBaSb might exhibit desirable charge- and heat-transfer properties that can make this material (and its analogs) a good candidate for exploring new thermoelectrics [33,34]. It can be speculated that the relatively large magnitude of the bandgap could be of advantage in circumventing a potentially detrimental bipolar conduction effects known in some candidate thermoelectric materials, thereby allowing the excitation of only the majority carrier. As alluded to already, the 3-D topological Dirac semimetal state in the hexagonal Na_3_Bi is well-established [35], and prior studies have indicated that its derivate, bismuthide NaBaBi (isostructural with NaBaSb), has evolved as a topological insulator (TI) through breaking of the inversion symmetry in Na_3_Bi with the characteristic Dirac cone surface states [32]. Thus, NaBaSb could also be an emerging topological insulator (TI).

**Figure 5:**
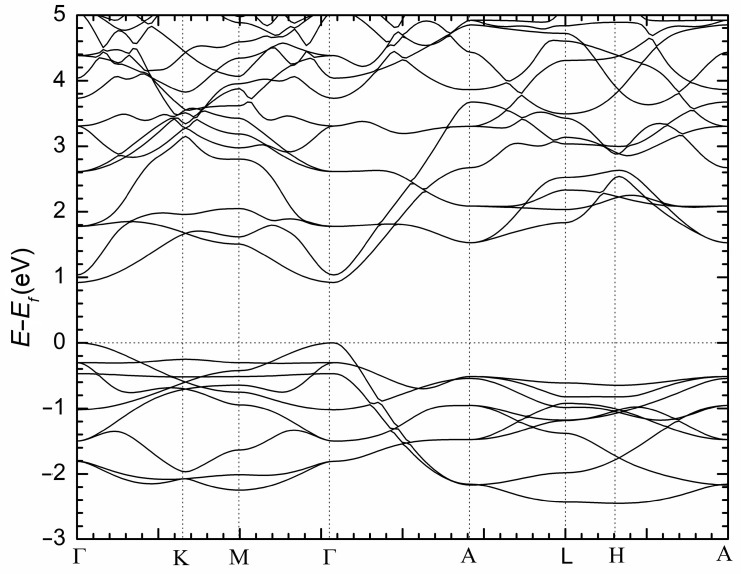
Electronic structure of NaBaSb.

Figure 5 shows the electronic band structure of NaBaSb in momentum space. As evident from the plot, there are no bands crossing from the valence band to the conduction band. The lowest energy separation between the top of the valence band and the bottom of the conduction band occurs at the Γ point. This observation is suggestive of a direct bandgap semiconductor with a potentially smaller than 0.5 eV bulk gap (vide supra). In addition, at the Γ point, the position and feature of the valence band maximum and the conduction band minimum show features that can be amenable to band inversion under strong SOC effect. Therefore, by drawing relevant ideas from the related phases that have been reported to show topological features, it is plausible to think of these phases as promising topological quantum materials. While the presented bulk electronic structure calculations are preliminary and without the inclusion of the spin–orbit coupling (SOC) effect, which is considered an essential ingredient that drives the realization of topological phases in condensed matter, the inclusion of SOC in such calculations that induce band inversion, as well as electronic structure calculation of the surface states, including the parity analysis, will be of great use to unambiguously classify these phases. We also note that in NaBaBi, the reported topological insulator phase was induced under applied pressure similar to the report of *A*In_2_As_2_ (*A* = Ca, Sr, Ba) Zintl phases [36,37]. If the prediction of the topological insulator phase can be considered for these compounds, the Eu-containing phase would likely present a profitable test material to explore possible higher-order topological insulator states [38].

Lastly, since it is a growing trend to explore the topological surface states of such materials to engineer high-performance thermoelectric materials, the well-defined band gap and band degeneracy in the representative phase suggest a great opportunity to explore these materials for such purposes.

## 3. Materials and Methods

The synthesis protocols for the exploratory work in the Na–*AE*–*Pn* systems mirrored those used for synthesizing the new Li–*AE*–*Pn* phases [16]. Briefly, all synthetic and post-synthetic manipulations were performed in an argon gas-filled glove box with O_2_/H_2_O levels below 1 ppm, or under vacuum. The elements were purchased from Alfa (Teksburry, MA USA)or Sigma-Aldrich (St. Louis, MO, USA) with stated purity 99.9 wt%. Sb shots were ground to powder, and the surface of the Na chunk was cleaned with a blade before cutting the metal into small pieces (immediately before use). On this note, we must reiterate the need to maintain an inert atmosphere every step of the way because most of the complications with the synthesis and the crystallographic studies arose from the extreme air and moisture sensitivity.

For the actual high-temperature reactions, the elements were placed in Nb tubes, which were then welded shut under argon. The closed Nb tubes were subsequently placed in silica tubes, which were then evacuated and flame sealed. The reactions were performed at 900 °C (ramp rate: 300 °C/h), for 24 h, cooled to 800 °C (rate –5 °C/h), annealed for 48 h, and subsequently air-quenched to room temperature. Then, the tube was brought back in a glove box and cut open.

Sodium reacts quickly with air, even more so at high temperature when the reaction could be violent. Caution must be exercised and to mitigate the risk, the silica jackets in such reactions should be sufficiently long that one of the ends of the tube protrudes outside the furnace—this must be performed for condensation of Na vapors in the event of a leak from the niobium ampoules into the silica tube. Additionally, one must be cognizant of the fact that Sb, and especially As, tends to react with Nb [39,40] which greatly exacerbates the risks.

X-ray powder diffraction data were obtained using a Rigaku MiniFlex (Rigaku Corporation, Tokyo, Japan) powder diffractometer, which was operated inside a nitrogen-filled glove box. Regardless of the steps taken to prevent contact of the samples with air and moisture, the X-ray powder diffraction patterns showed only high background and no Bragg peaks, which is an indication that the samples are extremely air-sensitive and/or may have decomposed in contact with the grease used during the experiments. All the characterization steps were performed via single-crystal X-ray diffraction.

The crystals were small and did not have well defined morphologies. Air stability was an issue (vide supra; therefore, single crystals were selected in the glove box (under a microscope) and cut to desired dimensions (around 0.1 mm) with a scalpel. This was easy because the crystals were very brittle. To handle the crystals prior to mounting them on the goniometer, they were covered in dry Paratone-N oil in the glove box and were quickly “scooped” from the oil droplet by using MitiGen plastic loops.

The intensity data were acquired using a nitrogen gas stream to alleviate the problem with air sensitivity. Temperature was maintained at 200 K throughout the experiments. Multiple crystals were tried before those of the best quality were identified. Intensity data were collected using a Bruker (Bruker AXS, Madison, WI, USA) SMART CCD diffractometer. The Bruker-supplied software packages [41,42] were used to manage data collection and for the integration of the measured reflections. Absorption correction was applied using SADABS [43]. Refinements by least-square minimizations on *F*^2^ were performed with the aid of the SHELXL program [44]. The atomic coordinates from the previous reports on the related NaBaBi [13] were used to create a starting model. Very reasonable conventional residual factors were obtained only after a few refinement cycles. Site occupancies were checked for each atom (for each structure) and no deviations from unit were found. In the last refinement cycles, the atoms were refined anisotropically, and TWIN and BASF instructions from SHELXL [44] were utilized in order to establish the correct absolute structures. This allowed us to achieve excellent fitting to the experimental data (Table 1). Final difference Fourier maps, in all cases, were featureless.

To calculate the electronic density of states (DOS) and band structure of NaBaSb, we used the Stuttgart TB-LMTO-ASA code [45] with the local density approximation. Experimental unit cell parameters and atomic coordinates for NaBaSb (Table 1 and Table 2) were used as the input parameters in our calculation. In order to satisfy the atomic sphere approximation (ASA), we employed von Barth-Hedin functional and introduced empty spheres to the calculation [46]. We employed 4 × 4 × 4 *k*-point grid for the Brillouin zone (BZ) integrations to accurately calculate the Fermi surface. The Fermi level was selected as the energy reference (*E*_F_ = 0 eV).

## Figures and Tables

**Table 1:** Selected single-crystal data collection and structure refinement parameters for NaSrSb, NaBaSb and NaEuSb. All three data sets were collected with Mo Kα radiation, *λ* = 0.71073 Å.

Empirical Formula	NaSrSb	NaBaSb	NaEuSb
Formula weight	232.36	282.08	296.70
Temperature (K)	200(2)	200(2)	200(2)
Space group, *Z*	*P*$\bar{6}$2*m*, 3	*P*$\bar{6}$2*m*, 3	*P*$\bar{6}$2*m*, 3
*a* (Å)	8.2183(3)	8.4779(4)	8.1514(4)
*c* (Å)	4.8475(4)	5.0338(5)	4.8154(5)
*V* (Å^3^)	283.54(3)	313.33(4)	277.09(3)
*c/a*	0.590	0.594	0.591
*ρ*_cal_ (g/cm^3^)	4.08	4.49	5.33
*μ* (cm^–1^)	210.9	156.9	239.7
Goodness-of-fit on *F*^2^	1.11	1.12	1.02
Unique reflections	329	383	355
Refined parameters	15	15	15
*R*_1_ (*I* > 2σ*_I_*) ^a^	0.0153	0.0173	0.0187
*wR*_2_ (*I* > 2σ*_I_*) ^a^	0.0330	0.0368	0.0361
*R*_1_ (all data) ^a^	0.0157	0.0179	0.0197
*wR*_2_ (all data) ^a^	0.0331	0.0369	0.0364
Largest peak and hole difference (*e*^–^/Å^3^)	0.84 and –0.85	0.61 and –0.89	0.88 and –0.82
CCDC deposition no.	2237818	2237817	2237816

^a^ *R*_1_ = ∑||*F*_o_| − |*F*_c_||/∑|*F*_o_|; *wR*_2_ = [∑[*w*(*F*_o_^2^ − *F*_c_^2^)^2^]/∑[*w*(*F*_o_^2^)^2^]]^1/2^, where *w* = 1/[*s*^2^*F*_o_^2^ + (*AP*)^2^ + (*BP*)] and *P* = (*F*_o_^2^ + 2*F*_c_^2^)/3. *A* and *B* are the respective weight coefficients (see the CIFs).

**Table 2:** Atomic coordinates of the atoms and their equivalent isotropic displacement parameters *U*_eq_^a^ for NaSrSb, NaBaSb and NaEuSb.

Atom	Site	*x*	*y*	*z*	*U*_eq_ (Å^2^)
NaSrSb					
Na	3*g*	0.2424(3)	0	1/2	0.0132(5)
Sr	3*f*	0.58090(8)	0	0	0.0146(2)
Sb2	2*d*	1/3	2/3	1/2	0.0119(1)
Sb1	1*a*	0	0	0	0.0151(2)
NaBaSb					
Na	3*g*	0.2400(4)	0	1/2	0.0173(8)
Ba	3*f*	0.58118(6)	0	0	0.0122(1)
Sb2	2*d*	1/3	2/3	1/2	0.0100(2)
Sb1	1*a*	0	0	0	0.0114(2)
NaEuSb					
Na	3*g*	0.2416(5)	0	1/2	0.0171(9)
Eu	3*f*	0.58180(7)	0	0	0.0142(1)
Sb2	2*d*	1/3	2/3	1/2	0.0125(2)
Sb1	1*a*	0	0	0	0.0132(2)

^a^ *U*_eq_ is defined as one third of the trace of the orthogonalized *U*_ij_ tensor.

**Table 3:** Selected interatomic distances (Å) in NaSrSb, NaBaSb and NaEuSb. Contacts longer than 4 Å are omitted.

Atom Pair	Distance (Å)	Atom Pair	Distance (Å)	Atom Pair	Distance (Å)
NaSrSb		NaBaSb		NaEuSb	
Na–Sb1 (×2)	3.138(2)	Na–Sb1 (×2)	3.237(2)	Na–Sb1 (×2)	3.111(3)
Na–Sb2 (×2)	3.180(2)	Na–Sb2 (×2)	3.293(2)	Na–Sb2 (×2)	3.158(3)
Na–Sr (×2)	3.690(2)	Na–Ba (×2)	3.834(3)	Na–Eu (×2)	3.672(3)
Na–Sr (×4)	3.8529(5)	Na–Ba (×4)	3.9822(5)	Na–Eu (×4)	3.8187(5)
Na–Na (×2)	3.451(4)	Na–Na (×2)	3.525(6)	Na–Na (×2)	3.412(7)
Sr–Sb1	3.4443(6)	Ba–Sb1	3.5508(6)	Eu–Sb1	3.4089(6)
Sr–Sb2 (×4)	3.4562(2)	Ba–Sb2 (×4)	3.5774(2)	Eu–Sb2 (×4)	3.4320(2)
Sr–Na (×2)	3.690(2)	Ba–Na (×2)	3.834(3)	Eu–Na (×2)	3.672(3)
Sr–Na (×4)	3.8529(5)	Ba–Na (×4)	3.9822(5)	Eu–Na (×4)	3.8187(5)
Sb1–Na (×6)	3.138(2)	Sb1–Na (×6)	3.237(2)	Sb1–Na (×6)	3.111(3)
Sb1–Sr (×3)	3.4443(6)	Sb1–Ba (×3)	3.5508(6)	Sb1–Eu (×3)	3.4089(6)
Sb2–Na (×3)	3.180(2)	Sb2–Na (×3)	3.293(2)	Sb2–Na (×3)	3.158(3)
Sb2–Sr (×6)	3.4562(2)	Sb2–Ba (×6)	3.5774(2)	Sb2–Eu (×6)	3.4320(2)

## Data Availability

The corresponding crystallographic information files (CIF) for all structures have been deposited with CSD, and the data for this paper can be obtained free of charge via https://www.ccdc.cam.ac.uk/structures/ (or from the CCDC, 12 Union Road, Cambridge CB2 1 EZ, UK; Fax: +44-1223-336033; E-mail: deposit@ccdc.cam.ac.uk). Depository numbers are 2237816–223818.

## Supplementary Materials

The following supporting information can be downloaded at: https://www.mdpi.com/article/10.3390/ma16041428/s1, Figure showing a structural representation of the similarities between the AlB_2_ and Fe_2_P (ZrNiAl) structures; table summarizing crystal data for the compounds formed between the alkali metals (*A*), the alkaline-earth metals (*AE*), and the group 15 elements P, As, Sb and Bi (*Pn*). References [47,48,49,50,51,52] are cited in the supplementary materials.
