# Supplementary material for: Synthesis and Crystal Structure of the Zintl Phases NaSrSb, NaBaSb and NaEuSb
Source: Materials (Basel). 2023 Feb 8;16(4):1428. doi: 10.3390/ma16041428 (PMC9959472; doi:10.3390/ma16041428)
Supplement: Supplementary file 1 [file materials-16-01428-s001.zip › materials-2210522-supplementary.pdf]

materials

MDPI

Supplemenaty Materials

Synthesis and Crystal Structure of the Zintl Phases NaSrSb, NaBaSb and NaEuSb

Yi Wang and Svilen Bobev *

Department of Chemistry and Biochemistry, University of Delaware, Newark, DE 19716, USA
* Correspondence: bobev@udel.edu

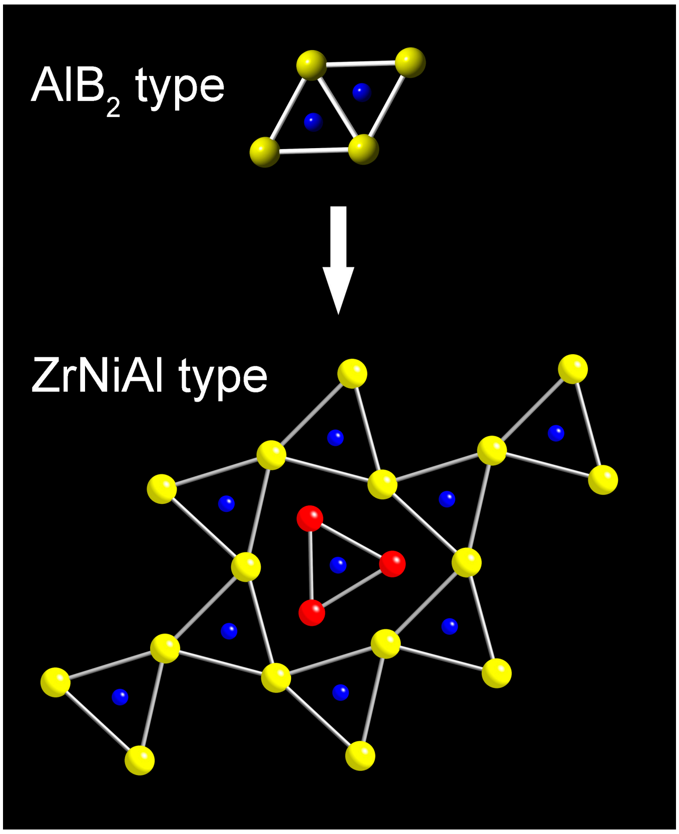

Figure S1. The hexagonal crystal structure of AlB_2, and the hexagonal crystal structure of ZrNiAl, both projected down the *c*-axis. The representation emphasizes the trigonal prisms having metal atoms at the corners and B atoms (AlB_2) and Al atoms ((ZrNiAl) at the center. The different three-dimensional connectivity of these prisms leads to the two structure types, respectively. For a comprehensive overview of the relationships between AlB_2-related intermetallic compounds, the reader is referred to the following review article: Hoffmann, R-D.; Pöttgen, R. AlB_2-related intermetallic compounds—a comprehensive view based on group-subgroup relations. *Z. Kristallogr.* **2001**, *216*, 127–145.

Table S1. Some known equiatomic compounds formed between the alkali metals (*A*), the alkaline-earth metals (*AE*), and the group 15 elements P, As, Sb, Bi, (*Pn*).

Compound	Structure Type	Space Group	Pearson Symbol	Ref.
LiBeP	PbClF (Cu_2Sb)	$P4/nmm$	$tP6$	[1]
LiBeAs	PbClF (Cu_2Sb)	$P4/nmm$	$tP6$	[2]
NaMgAs	PbClF (Cu_2Sb)	$P4/nmm$	$tP6$	[3]
NaMgSb	PbClF (Cu_2Sb)	$P4/nmm$	$tP6$	[3]
NaMgBi	PbClF (Cu_2Sb)	$P4/nmm$	$tP6$	[4]
KMgP	PbClF (Cu_2Sb)	$P4/nmm$	$tP6$	[5]
KMgAs	PbClF (Cu_2Sb)	$P4/nmm$	$tP6$	[5]
KMgSb	PbClF (Cu_2Sb)	$P4/nmm$	$tP6$	[5]
KMgBi	PbClF (Cu_2Sb)	$P4/nmm$	$tP6$	[5]
KCaBi	PbClF (Cu_2Sb)	$P4/nmm$	$tP6$	[6]
RbCaAs	PbClF (Cu_2Sb)	$P4/nmm$	$tP6$	[7]
RbCaSb	PbClF (Cu_2Sb)	$P4/nmm$	$tP6$	[7]
LiMgP	LiAlSi (half-Heusler)	$F\bar{4}3m$	$cF12$	[1]
LiMgAs	LiAlSi (half-Heusler)	$F\bar{4}3m$	$cF12$	[8]
LiMgBi	LiAlSi (half-Heusler)	$F\bar{4}3m$	$cF12$	[8]
LiSrP *	LiBaSi	$P\bar{6}m2$	$hP3$	[9]
LiBaP *	LiBaSi	$P\bar{6}m2$	$hP3$	[9]
LiBaAs	LiBaSi	$P\bar{6}m2$	$hP3$	[9]
LiBeSb	LiGaGe	$P6_3mc$	$hP6$	[10]
NaBeAs	ZrBeSi (KZnP)	$P6_3/mmc$	$hP6$	[11]
NaBeSb	ZrBeSi (KZnP)	$P6_3/mmc$	$hP6$	[11]
LiSrP *	ZrBeSi (KZnP)	$P6_3/mmc$	$hP6$	[12]
LiBaP *	ZrBeSi (KZnP)	$P6_3/mmc$	$hP6$	[12]
LiBaSb	ZrBeSi (KZnP)	$P6_3/mmc$	$hP6$	[13]
CaLiAs	TiNiSi (SrMgSi)	$Pnma$	$oP12$	[14,15]
CaLiSb	TiNiSi (SrMgSi)	$Pnma$	$oP12$	[14,15]
CaLiBi	TiNiSi (SrMgSi)	$Pnma$	$oP12$	[14,15]
SrLiAs	TiNiSi (SrMgSi)	$Pnma$	$oP12$	[16]
SrLiSb	TiNiSi (SrMgSi)	$Pnma$	$oP12$	[14,17]
SrLiBi	TiNiSi (SrMgSi)	$Pnma$	$oP12$	[14]
NaSrP	ZrNiAl (Fe_2P)	$P\bar{6}2m$	$hP9$	[12]
NaSrAs	ZrNiAl (Fe_2P)	$P\bar{6}2m$	$hP9$	[18]
NaBaP	ZrNiAl (Fe_2P)	$P\bar{6}2m$	$hP9$	[19]
NaBaBi	ZrNiAl (Fe_2P)	$P\bar{6}2m$	$hP9$	[20]
$SrLi_{0.95}As_{0.98}$	-	$P6_3/mmc$	-	[16]

* contradicting structure determinations.

References

1. El Maslout, A.; Motte, J.P.; Gleitzer, C.; Aubry, J. Preparation et proprietes d'un nouveau compose dans la serie LiMP: le phosphure de lithium-cadmium LiCdP. *Comptes Rendus Des Seances De L'academie Des Sci. Ser. C: Sci. Chim.* **1971**, *273*, 707–710.
2. Tiburtius, C.; Schuster, H.U. LiBeSb und LiZnBi, ternaere Verbindungen mit Wurtzitgeruest. *Z. Naturforsch.* **1978**, *33b*, 35–38.
3. Krenkel, B.; Schuster, H.U. NaMgAs(Sb)—Ternaere Verbindungen mit modifizierter Cu_2Sb-Struktur. *Z. Naturforsch.* **1978**, *33b*, 1080–1082.
4. Yamada, T.; Matsuo, N.; Enoki, M.; Yamane, H. A novel ternary bismuthide, NaMgBi: crystal amd electronic structure and electrical properties. *Z. Naturforsch.* **2021**, *76b*, 789–795.
5. Vogel, R.; Schuster, H.U. Neue elektrovalente ternare Verbindungen des Kaliums mit Magnesium unde Elementen der 5 Hauptgruppe. *Z. Naturforsch.* **1979**, *34b*, 1719–1721.

6. Hirt, H.; Deiseroth, H.J. Crystal structure of potassium calcium bismuthide KCaBi. *Z. Kristallogr. NCS* **2003**, *218*, 191
7. Cardoso, G.; Caroca-Canalesr, N.; Hönle, W.; von Schnering, H.-G. Crystal structure of rubidium calcium arsenide, RbCaAs, and rubidium calcium antimonide, RbCaSb. *Z. Kristallogr. NCS* **2003**, *218*, 455–456.
8. Nowotny, H.M.; Holub, F. Untersuchungen an metalischen Systemen mit Flussspatphasen. *Monatsh. Chem.* **1960**, *91*, 877–887.
9. Albering, J.H.; Ebel, T.; Jeitschko, W. Praeparation, Kristallstruktur und magnetische Eigenschaften der Verbindungen Li*AX* (*A* = Ca, Sr, Ba, Eu, Yb; *X* = P, As, Sb, Bi). *Z. Kristallogr. Supple. Issue* **1997**, *12*, 242.
10. Tiburtius, C.; Schuster, H.U. LiBeSb und LiZnBi, ternaere Verbindungen mit Wurtztigeruest. *Z. Naturforsch.* **1978**, *33b*, 35–38.
11. Tiburtius, C.; Schuster, H.U. NaBeAs(Sb) – ternaere Phasen im 'aufgefuellten' NiAs (Ni_2In)-Typ. *Z. Naturforsch.* **1978**, *32b*, 1133–1138.
12. Dong, Y. K.; DiSalvo, F.J. Synthesis and single crystal structures of ternary phosphides Li_4SrP_2 and *AAe*P (*A* = Li, Na; *Ae* = Sr, Ba). *J. Solid State Chem.* **2007**, *180*, 432–439.
13. Monconduit, L.; Belin, C. A new ternary antimonide phase, LiBaSb. *Acta Cryst. E.* **2001**, *57*, 17–18.
14. Eisenmann, B.; Liebrich, O.; Schäfer, H.; Weiss, A. Darstellung und Kristallstruktur von CaLiSb (Ternaere E-Phasen von Hauptgruppenelementen II). *Z. Naturforsch.* **1969**, *24b*, 1344–1345.
15. Schäfer, M. C.; Suen, N.-T.; Bobev, S. Synthesis and crystal chemistry of new ternary pnictides containing lithium—adding structural complexity one step at a time. *Dalton Trans.* **2014**, *43*, 1688.
16. Feng, X.J.; Prots, Y.; Schmidt, M. P.; Hoffmann, S.; Schnelle, W.; Burkhardt, U.; Zhao, J.-T.; Grin, Y. Synthesis, structure, and properties of two Zintl phases around the composition SrLiAs. *Inorg. Chem*. **2013**, *52*, 8971–8978.
17. Gupta, S.; Ganguli, A.K. Synthesis, structure and properties of a new Zintl phase: SrLiSb. *J. Soild State Chem.* **2006**, *179*, 1318–1322.
18. Carrillo Cabrera, W.; Somer, M.; Peters, E.M.; Peters, K.; von Schnering, H.-G. Crystal structure of sodium strontium arsenide, NaSrAs. *Z. Kristallogr.* **1997**, *212*, 252.
19. Carrillo Cabrera, W.; Somer, M.; Peters, E.M.; Peters, K.; von Schnering, H.-G. Crystal structure of sodium barium phosphide, NaBaP. *Z. Kristallogr.* **1997**, *212*, 191.
20. Hirt, H.; Deiseroth, H.J. The new polar intermetallic compound NaBaBi. *Z. Anorg. Allg. Chem.* **2004**, *630*, 1357–1359.

Disclaimer/Publisher's Note: The statements, opinions and data contained in all publications are solely those of the individual author(s) and contributor(s) and not of MDPI and/or the editor(s). MDPI and/or the editor(s) disclaim responsibility for any injury to people or property resulting from any ideas, methods, instructions or products referred to in the content.
